# Physical activity advice given by French general practitioners for low back pain and the role of digital e-health applications: a qualitative study

**DOI:** 10.1186/s12875-024-02284-w

**Published:** 2024-01-29

**Authors:** Marion Dehainault, Olivia Gaillard, Bintou Ouattara, Matthieu Peurois, Cyril Begue

**Affiliations:** 1 Faculty of Health, General Practice Department, University of Angers, Angers, F-49000 France; 2 Univ Angers, CHU Angers, Univ Rennes, EHESP, Irset (Institut de Recherche en Santé, Environnement et Travail) - UMR_S 1085, IRSET-ESTER, SFR ICAT, CAPTV CDC, Inserm, Angers, F- 49000 France

**Keywords:** Low back pain, Physical activity, General medicine, Physiotherapy, Mobile applications, e-health, France, New Technologies for Information and Communication

## Abstract

**Background:**

Low back pain is the fourth most common reason for consulting a general practitioner (GP) among people aged 40–50 years. Beyond the overall benefits of physical activity (PA) on health (psychological, cardiovascular, etc.), PA for low back pain seems to improve the prognosis in terms of pain, disability, and quality of life. The French National Health Insurance developed media campaigns to promote physical activity with low back pain and a smartphone application (app). Despite the known benefits and campaigns, GPs do not routinely provide advice about physical activity during low back pain consultations. To promote giving physical activity advice for low back pain, there is a need to understand how GPs currently provide this advice and whether technology could help. This study aims to explore the content of physical activity advice for low back pain that GPs provide in France, and their opinion about healthcare smartphone app provided electronically via the internet (e-health apps) as a support for this advice.

**Methods:**

This qualitative study was conducted with semi-structured individual interviews among French GPs. The verbatim was double coded using a coding tree. Thematic analysis was performed using an inductive approach.

**Results:**

Sixteen GPs from Maine et Loire, Sarthe, and Mayenne were included. The thematic analysis identified the following themes: GPs use a global patient-centred approach to physical activity advice for low back pain. The main goal is to enable patients to participate in their care. Advice was almost always general with little information about duration and frequency. The importance of patient-appropriate and easily achievable activities was emphasised. GPs referred patients to physiotherapists to reinforce regular physical activity, maintain motivation and improve patient adherence through supervision and follow-up. GPs knew little about e-health apps but felt they could be useful with young patients. The main barriers to their use included poor internet connection, lack of technical knowledge and no supervision meaning patients could injure themselves.

**Conclusions:**

This is one of the first studies to assess the contents of physical activity advice GPs provide for low back pain. Further research is needed into the implementation of e-health apps for low back pain management.

**Trial registration:**

Not applicable.

**Supplementary Information:**

The online version contains supplementary material available at 10.1186/s12875-024-02284-w.

## Background

Low back pain is the fourth most common reason for consulting a general practitioner (GP) in people aged between 40 and 50 years [1] and accounts for approximately 6 million consultations in France each year [2]. Furthermore, low back pain accounts for 20% of workplace accidents and 7% of occupational illnesses with an estimated cost of more than one billion euros annually [3].

Beyond the overall benefits of physical activity (PA) on health (psychological, cardiovascular, …), PA in low back pain seems to improve prognosis in terms of pain, disability, and quality of life and feature in National Health Authority (HAS) recommendations in France [4], Europe, the United States, England, and Canada [5–7]. However, it was shown that a majority of GPs in England are unfamiliar with the national PA guidelines [8].

Promoting PA for health is becoming commonplace around the world. Firstly, Finland, the United Kingdom, and the Netherlands developed national strategies to promote PA for health [9]. Also, the World Health Organization (WHO) and Health Enhancing Physical Activity (HEPA) Europe also work to promote health through sport [10, 11]. Furthermore, the French National Health Insurance developed specific PA media campaigns for low back pain and a smartphone application (app) called “Activ’Dos” [12–14].

Digital and e-health tools offer new ways to help patients. To be beneficial, these tools must be based on reliable information, updated regularly, involve competent professionals with no conflict of interest be easy to use, confidential, and protect personal data [15]. Currently, few low back pain apps available in France meet all the expected quality criteria. Firstly, “Mon Coach Dos” was developed with the physical medicine and rehabilitation department of Clermont-Ferrand University Hospital and doctors specialising in physical medicine and rehabilitation, physiotherapists, occupational therapists and adapted physical activity (APA) trainers [2]. Subsequently, “Activ’dos” and “Doado”, a similar app, were released which provide information about low back pain, lifestyle advice, home-based exercises and answer frequently asked questions [16].

Despite these recommendations and GPs understanding its importance, PA advice is not routinely given during low back pain consultations and dedicated PA consultations are rarely provided [17]. This may be due to perceived obstacles such as patients needing encouragement, education, access to information and support to involve them in their disease management [2]. Furthermore, the available literature on which activity type, frequency, duration, and intensity are appropriate for low back pain is often of low quality with insufficient detail, meaning advice is vague, with no demonstrated superiority of one activity over another [4, 6, 18, 19]. In addition, little is known about the advice GPs provide since qualitative studies in this area are rare and are mostly those with small sample sizes [20].

To improve the promotion of PA for low back pain, there is a need understand how GPs currently provide PA advice to their patients and whether technologies could help. This study therefore aims to explore the content of PA advice for low back pain that GPs provide in France, and their opinion about healthcare smartphone app provided electronically via the internet (e-health apps) as a support for this advice.

## Methods

This qualitative study was conducted using semi-structured individual interviews with GPs from Maine et Loire, Sarthe, and Mayenne, France. Eligible GPs working in these regions were identified and recruited from the regional lists of the national GP register. GPs practising general medicine in a primary care practice were eligible for inclusion. Physicians not practising general medicine and locum GPs were excluded. All GPs were contacted directly by telephone or e-mail or through their secretary. Purposive sampling was used to obtain maximum variation thus ensuring data was collected from a wide range of perspectives. GPs were recruited in order to obtain variation in the following characteristics: age, gender, number of years working at their current practice, place of practice (rural, semi-rural, urban) and whether they were a university internship tutor. Interviews were conducted until data saturation. The interviews were stopped when it became clear that the data received was being repeated and that no new data was emerging. Two additional interviews were conducted as a precautionary measure to ensure that this was the case.

### Data collection

MD and OD, two female, MD students who had received training in interview techniques conducted the interviews between December 2, 2020, and February 26, 2021. GPs were offered a remote or face-to-face interview in their chosen location. Only one interviewer and the participating GP were present during the interviews. Participants were aware they would be interviewed about the content of PA advice for patients with low back pain but were not informed that e-health apps would be discussed so they were not prepared. They were also informed that this research was for the interviewers’ medical doctoral theses. The two interviewers did not know the participating GPs prior to the interviews. Field notes were taken during each interview.

The interview guide was developed based on literature data and contained five open-ended questions with follow-up questions depending on the type of response given. Following the first and seventh interviews, the guide was modified to refine the follow-up questions (Appendix I). A slideshow presenting screenshots from the “Mon Coach Dos” and “Activ’Dos” mobile apps was used with a standardised explanatory presentation framework. An information sheet about the apps was integrated into the slideshow presenting their description, creator, funding, and data use (Appendix II). The “Doado” app was withdrawn from the study before the first interview due to technical problems related to its use.

The interviews were audio recorded after participants provided informed written consent. The content was then fully transcribed verbatim on Microsoft Word© ensuring anonymity.

### Data analysis

Thematic analysis was performed using an inductive approach. The two researchers familiarised themselves with the data then each researcher carried out open coding per meaning unit. The two researchers jointly grouped the meaning units depending on how they related to the theme and their link to the research question. This iterative grouping approach created a thematic tree using triangulation. Any discrepancies were discussed and if no consensus was reached, a third investigator (CB) was consulted. NVivo© software (Version 12) was used for data collection and analysis. During the interview, participants were asked if they would like to receive the transcript of the interview to verify their statements. When the results were finalized, an email was sent to them asking if they would like to receive the full manuscript before submission. No participant requested receipt of the transcript and/or manuscript.

### Ethics

The study received approval from the Angers University Ethics Committee (2020/20).

## Results

In total, 16 GPs were included, of which nine were female and seven were male, with an average age of 42.75 years [29–64 years]. Eight GPs practised in Maine et Loire, four in Sarthe and four in Mayenne. The interviews were all conducted face-to-face and lasted an average of 42 min. Eight GPs worked in a rural area, four in a semi-rural area and four in an urban area. Nine GPs were university internship tutors and one had training in sports medicine and osteopathy (Table 1).The thematic analysis identified the themes and subthemes shown in Table 2.

**Table 1:** Simplified chart of sample characteristics

GP	Gender	Age	Years of Installation	Practice
1	F	51 yo	17 years	Rural
2	F	53 yo	20 years	Urban
3	M	29 yo	3 months	Rural
4	F	43 yo	19 years	Rural
5	M	64 yo	30 years	Urban
6	F	38 yo	6 years	Rural
7	M	51 yo	20 years	Urban
8	M	32 yo	2 years and a half	Rural
9	F	64 yo	32 years	Semi-Rural
10	F	42 yo	13 years	Rural
11	M	46 yo	11 years	Rural
12	M	34 yo	3 years	Semi-rural
13	F	31 yo	1 year	Rural
14	F	30 yo	18 months	Urban
15	M	39 yo	7 years	Semi-rural
16	F	37 yo	7 months	Semi-rural

Yo = years old

**Table 2:** Hierarchical table of themes and subthemes

**I. GPs discuss PA with their low back pain patients**
a. General advice
b. Advice tailored to their patients
c. Advice focused on their experiences (patients and doctors)
d. Barriers to the practicing PA
**II. Use of physiotherapists**
a. GPs judge them to be better trained on giving PA advice
b. Active and safe work
c. Tailored and reproducible exercises
d. Supervision that promotes patient compliance
e. Accessibility issues
**III. Mobile applications are little known to GPs**
a. Connected tools that are part of the evolution of society and health
i. Reliable and complete content
ii. Ease of accessibility
iii. Strengthening medical discourse
iv. Motivational help for the patient
b. The obstacles to their use according to the MG

Almost all GPs described their patients with low back pain as having a physically demanding job, often carrying heavy objects, or excessively straining the lumbar region. Their low back pain sometimes affected daily and professional life with frequent need for time off work. Due to the multifactorial nature of low back pain, GPs generally described having a global approach to management taking into account the biomedical and psychosocial aspects. Some GPs mentioned that treating practises have changed over the past twenty years with movement being promoted rather than rest. Most participating GPs felt that most GPs and patients recognised the benefits of PA.

### General practitioners and physical activity advice for low back pain

#### Advice content

Almost all participating GPs said they did not provide detailed advice, instead they gave general advice about PA: *“It’s true that I don’t prescribe it like a medicine; so many times a day for a particular length of time. It’s a bit of general advice that will be added to everything else”* (GP2). Some reported not having *“the skills”* (GP11) or not being *“an expert”* (GP9).

Most GPs recommended moving in any way and *“not staying on the sofa or in bed”* (GP4). During the acute pain phase, most GPs advised patients to move but without forcing it: *“I tell them that you have to have physical activity called relative rest. That is to say, do things that don’t hurt.”* (GP16). They recommended walking since it seems to be easier: *“walking is more of a physiological thing so it might already be easier to do”* (GP2) and it *“costs nothing to do, does not require equipment”* (GP14). They also recommended swimming *“when it’s possible, I recommend swimming. I think it’s really good, it has good virtues, and it helps strengthen the back quite a bit”* (GP12). Other activities discussed included running, water aerobics, rowing, cycling, or stretching exercises such as gymnastics or yoga. When the gym was mentioned, GPs recommended being *“accompanied by a trainer”* (GP11). Some GPs reported prescribing adapted physical activity (APA) for *“patients with low back pain and a long-term condition”* (GP8). During the acute pain phase, some participating GPs discouraged certain activities such as carrying heavy loads, impact sports, and high intensity exercise: *“There are a lot of young people who push themselves a little too much; teenagers, young adults who play sports intensively. I think they are going too hard. They’re going too fast. […] They want to perform. They want to be the best, the fastest. There are 4 or 5 of them pedalling, doing I don’t know what, lifting heavy weights and then they want to be the best, they’re showing off”* (GP5).

Advice about PA duration and frequency was not always given. However, when participating GPs did provide this advice, duration ranged from 30 min to 1 h and frequency ranged from daily to weekly. Most GPs agreed that the most important instruction was to perform PA regularly. However, some GPs advocated a gradual recovery.

Most GPs gave advice about posture including carrying heavy loads where applicable and some demonstrated how to do the exercise or the movement: *“I get up and show them how to bend their legs with a straight back”* (GP14). In contrast, others gave general oral explanations or relied on written advice to give patients motivation and encourage involvement in their management.

Most GPs had no support materials to give patients. Those who used them mentioned referring patients to exercise videos, information on the internet, exercise sheets provided by laboratories, the National Health Insurance booklet or website, or their own materials. *“I often start with my file where I have many exercises and I mark those I think they can do”* (GP1). Some offered flyers for associations or sporting events such as a walk offered by a walking club, aquagym classes, an introduction to a yoga class, participation in village sports days, flyers concerning a number of free months in a gym, etc.: *“For example, next to the stadium, you have like a hundred different activity clubs who come, who set up a small stand, who present their stuff and where you can try different sports”* (GP8).

The long-term objective for most participants was to encourage patients to be active and involved in their care through regular exercise: *“Because the problem is in the long term, that’s what I tell people, you mustn’t do a little now and then do nothing”* (GP9). Participants highlighted various strategies that they used (Table 3).

**Table 3:** Strategies GPs use to encourage patients to be active and involved in their care

Strategies	Illustrative quotes
Exercising with others to improve motivation and adherence	*“When patients tell me they exercise with their husband or their neighbour I know that they will probably be more committed as it’s a shared thing. It’s pleasant, they have a connection and they’re not walking on their own”* (GP6)
Enjoying different activities that are not restrictive, easy to set up, and accessible, while avoiding injuries through gradual recovery	*“The activity must vary because otherwise they get bored, they give up (…) they often set goals that are too aggressive, too fast and they get discouraged”* (GP8)
Changing everyday habits	*“Parking further away, walking, taking the stairs”* (GP10) *“Don’t take the car, use a bike. Walk or use public transport. Don’t use the elevator, take the stairs. Walk the dog. Do your shopping on foot if it’s for a baguette and not very far”* (GP14)
Giving patients choices to improve adherence	*“If you impose something on them, it won’t work so well. Patients will choose according to who they are, their environment, and their lifestyle”* (GP9)
Stopping patients from feeling guilty if they don’t achieve their goals	*“Patients should do as well as they can”* (GP8)
Prescribing physical activity, in particular APA	*“Sometimes when we have the chance, I don’t know if it’s luck, but if they have lower back pain and diabetes I’ll give them a prescription to play sports. Like that it’s prescribed by the Doctor, I don’t know why but when it’s prescribed by the doctor it’s always more motivating” (GP8)*

GP: General Practitioner

Some GPs reported that PA is also a useful way to avoid low back pain and prevent flare-ups in patients with chronic low back pain: *“it’s important to do more physical activity because that’s what will help their backs. I explain to them that a back which is not muscular is more vulnerable. The idea is prevention”* (GP14).

### Personalising physical activity advice

Almost all GPs highlighted the importance of giving PA advice for recurrent or chronic low back pain, often trying to motivate patients at each consultation but that’s not all: *“repeating, saying it in another way, then repeating at the next consultation. You can’t motivate someone in a single session. […] Just because we say it once doesn’t mean it’s going to happen. But I think if we repeat it, we repeat it, we repeat it, maybe”* (GP13). However, some GPs preferred waiting to discuss PA until the acute pain phase had improved: *“When a patient can’t move, they’re stuck in bed, it’s not the right moment to say ‘Okay, we have to move now’. Pain relief is the most important thing at first, but I do think the faster the patient can move, the better”* (GP7).

#### Patient-centred approach

Nearly all participating GPs discussed the factors they considered when personalising PA for each patient such as patient preference, pain, age, and lifestyle (Table 4). Other criteria are more inherent to the GP’s judgment. For example, some GPs discussed patient feedback and how this helps to *“highlight what has been effective”* (GP14). Others described being able to *“tell when the patient is not motivated so not insisting (…) it could frustrate them or make them feel devalued”* (GP8). GP knowledge of their patients was also mentioned as participants felt they *“generally know which patients are already active and which ones need encouragement to exercise”* (GP1).

**Table 4:** Patient factors GPs consider when personalising PA advice for patients from most to least commonly reported factors

Patient factors	Illustrative quotes
Patient likes and dislikes	*“You need to try to find a physical activity that is both good for them and that they like”* (GP15)
Pain, experience, and apprehensions	*“If a patient tells me it hurts when they walk, I can be sure they aren’t going to do it” (GP6)*
The PA the patient already does and their abilities	*“I ask if they do sports, which some patients already do on a daily basis.”* (GP9)
Age	*“It depends a bit on the age and the person. I often recommend walking on flat ground, swimming, and cycling. Among the younger patients, I recommend Pilates”* (GP4)
How open the patient is to change	*“It is important to judge when a patient is ready to change, wants to hear about options, or wants to get better”* (GP11)
Comorbidities	*“When you see a patient with acute low back pain and another condition such as hypertension, diabetes or obesity, you have to approach them differently”* (GP13)
Lifestyle and associated personal and professional constraints	*“I encourage them to do it every day. But if they can’t, I tell them to do it every other day or when they have time”* (GP8)
Season	*“Patients are more motivated to go walking in the spring but in the winter, patients prefer to exercise at home”* (GP8)

PA: Physical Activity; GP: General Practitioner

#### GP knowledge

One of the participating GPs had a background in sports medicine and osteopathy, and another in functional rehabilitation meaning they felt comfortable giving PA advice. However, most of the other GPs reported giving advice based on their personal and professional PA experience such as *“advising patients to do some of the stretches I do at taekwondo”* (GP12).

Some participants described information sources they used. This included scientific reviews and studies showing that *“physical activity is linked to life expectancy”* (GP11). Others mentioned following *“recommendations from cardiology societies which state 30 minutes of physical activity per day”* (GP14). Some described *“sessions about physical activity for low back pain and sessions with physiotherapists who are focused on exercise, strengthening and stretching”* (GP6). Textbooks, congresses, continuing professional development, personal research, and discussions with peers were also cited.

### Barriers to giving physical activity advice and exercising

The patient factors that GPs reported using to personalise PA advice (Table 4) can occasionally be barriers. In addition to these, GPs reported many other patient-related barriers (Table 5).

**Table 5:** Patient-related barriers to GPs giving physical activity advice and patients exercising from most to least cited barrier

Barriers	Illustrative quotes
COVID-19 pandemic and its consequences including closure of sports facilities, health restrictions and the psychological impact	*“Next door we have the swimming pool and gym. (…) plenty of patients have not gone back there because they were afraid of Covid 19”* (GP8)
Cost of physical activity, particularly sports club memberships	*“I am not sure that everything has to be free. Memberships are not very expensive”* (GP13)
Lack of time, which some participating GPs felt was an excuse	*“It’s up to them if they want to make the time but there is always half an hour to spare”* (GP13)
Patient beliefs with some believing you should rest not exercise when you have low back pain	*“Often, they mention cleaning, gardening, DIY, their job. But they don’t make any progress, it’s irregular”* (GP14).
Lack of suitable sports facilities, remoteness of some patient’s homes, and lack of infrastructure such as fitness trails or cycle paths, particularly in rural areas	*“We don’t have a local gym, so patients have to travel 25 km to go to the gym and that is very difficult”* (GP11) *“There are very few bike paths and the roads can be dangerous on a bike. There are people who like cycling but don’t do it because of this”* (GP15)
Fatigue, particularly associated with a working day	*“They work and are tired and there is nothing we can do about that”* (GP11)
Patient expectations	*“Sometimes we have the impression that they expect us to deliver them from evil”* (GP16)
Patient excuses	*“They exercise initially but they won’t walk in winter because there could be ice. They won’t go when it’s too dark, foggy or raining”* (GP8)
Sedentary culture, particularly the addiction to screens	*“How do we make physical activity more attractive than video games?”* (GP15)

PA: Physical Activity; GP: General Practitioner

The most reported GP-related barrier was lack of knowledge and training about PA, with some participants stating that *“If we were better trained, we would be more successful at passing on advice about prevention”* (GP13). However, some GPs reported that *“In practice, even if I had more precise information, I’m not sure I would change my discourse as a general practitioner since I already insist on the fact that it is important for everything, not just for the back but for everything. For all metabolic diseases. To relax. For stress, I already bring up the subject of physical activity a lot, I would say. It’s not sure that I would go further if I knew more”* (GP16). In addition, some GPs mentioned the lack of time during consultations meaning they are *“forced to get to the point”* (GP2): *“consultations are full in general medicine: the health and diet rules, all the prescriptions, the examination, the interview which is a little long too. Especially if there are psychological factors. This ends up making a fairly banal and very frequent consultation during the week into a fairly full consultation load. So that’s more of it. But I think that’s the bane of all doctors: lack of time for everything”* (GP14).

### Using physiotherapists as physical activity experts

Almost all participating GPs discussed referring patients to physiotherapists without being specifically questioned about this during the interviews. They described being able to refer to physiotherapists “*easily”* (GP14) and *“quickly”* (GP15). Most GPs felt that physiotherapists are better trained to give PA advice because *“it is part of their job”* (GP9) and they are *“more experienced at demonstrating exercises and postures”* (GP2). Most often, the physiotherapy referral was not detailed and was left to the physiotherapist’s discretion.

GPs described different reasons for physiotherapy referral. Some GPs reported that *“With patients who do not exercise regularly, I would rather refer them to the physiotherapist because it will force them to do it”* (GP5). Others described physiotherapist referral as a *“form of exercise coaching”* (GP2) particularly for patients *“who are not at all motivated, those who don’t do any sports, or who don’t feel like getting into it”* (GP2). It was also mentioned that *“When a patient has recurrent low back pain, works in a physically demanding job, or has very significant levels of pain, I will quickly refer them to the physiotherapist”* (GP15).

The benefits of physiotherapist referral included patients being able to *“exercise at home”* (GP2), the ability of physiotherapists to adapt PA to existing comorbidities, and the *“follow-up”* (GP16) physiotherapists provide. In addition, some participants reported that physiotherapy referral promotes regular PA, maintains motivation for PA resumption or continuation, and encourages patients through the *“competitive spirit”* (GP15) created during group PA in some rehabilitation programmes.

However, a large proportion of participants reported difficulties accessing physiotherapists due to long waiting lists or lack of physiotherapists close to their practice. In contrast, some participants reported no accessibility concerns thanks to the number of professionals, multidisciplinary centres, and multi-professional protocols. One GP stated that *“the practice is set up for low back pain with the physios. When low back pain risks becoming chronic, we provide a physiotherapist appointment within the week”* (GP13).

### Knowledge and opinions about mobile e-health apps

The participants knew very little about mobile e-health apps, but some recognised the logos of the organisations behind the applications (Health Insurance and Thuasne) during the slideshow. All participants were positive about recommending these apps as an additional support. However, before recommending them, they felt they would need time getting comfortable using these new technologies, *“seeing how they work”* (GP1) and *“what it can provide the patient”* (GP7) but this would *“take time and require the GP to be interested in the app”* (GP14).

Participating GPs described e-health apps as a *“tool that fits with the time”* (GP5) especially since *“almost everyone has a smartphone”* (GP3). Most GPs felt that the free and instant access to the app was beneficial since it could help patients to start exercising while waiting for an appointment with the physiotherapist: *“It’s not at all the same care you get from a physiotherapist, but at least it gets the patient moving”* (GP6). GPs highlighted that the described exercises can be done at any time, alone, and at home, and require little or no equipment enabling patients to *“realise that there are many exercises you can do at home”* (GP4).

Some GPs highlighted the *“complete”* (GP4) and *“exhaustive”* (GP11) information the apps provide including anatomical reminders, exercise demonstrations and postural advice: *“When patients have questions, the apps seem sufficiently comprehensive so that patients can find some answers”* (GP11). For this reason, participants felt e-health apps could be a complementary and additional support to reinforce PA advice: *“It is a useful aid to bring the patient back to thinking about his condition and putting it into practice”* (GP5). In addition, some GPs felt that the apps could improve motivation and *“empower patients”* (GP11) making them a participant in their care: *“Anyway, when we are more supervised in general there is more of an effect. […] If you are sensitive to that, it must help you. More than a doctor that you see once every so often. It maintains motivation so it gives more effect that’s for sure. […] They are completely involved in their pain and their muscle strengthening.”* (GP6). The app was described as *“creating a global approach to care and improving care continuity. The medical discourse is coherent, and the patient feels cared for”* (GP5). GPs also mentioned the *“COVID compatibility”* (GP6) of the apps as they can be used during lockdowns or when sports facilities are closed. However, most GPs felt that e-health app use would be limited to young, connected, and motivated patients with chronic low back pain. A range of potential barriers to their use were discussed (Table 6).

**Table 6:** Potential barriers to e-health app use

Potential barriers to e-health app use	Illustrative quotes
Poor internet connection, particularly in rural areas	*“There are more people than you think in our region who don’t have internet access”* (GP11)
Lack of technology skills	*“In our region, there are a lot of young people who do not have the skills, contrary to what we believe”* (GP11)
Sociocultural disparity with some patients not owning a smartphone or tablet	*“There are a lot of young people who don’t have a smartphone”* (GP11)
Elderly patients who use little or no technologies	*“It’s assumed that people have a certain mastery of computers but that’s not necessarily the case for everyone (…) I’m talking about people of a certain age”* (GP1) *“Some patients won’t have the reflex to check their tablet all the time. Although there are those who are hyper connected”* (GP7)
Lack of patient motivation and adherence	*“You have to have the courage to exercise alone”* (GP9) *“Those under 35 won’t do it much. More out of laziness.”* (GP8)
Inability to personalise to the patient so inappropriate use possible in the event of comorbidities	*“See this [shows an exercise on the app]. It may be good for low back pain but for a patient who had shoulder pain, I would advise against it”* (GP5)
Lack of supervision meaning patients don’t receive feedback to correct how they are doing the exercises and thus prevent injuries	*“There is no feedback. Patients follow images. Some people know how to reproduce and understand the movements that are shown to them but there are others who do it very badly and don’t know how to correct themselves”* (GP15)
Lack of human contact	*“It would not suit me at all. I would have preferred to see someone in real life”* (GP6) *“I don’t want to be pessimistic but doing everything alone is hard, you still need motivation otherwise, people get bored. Being alone can be a barrier”* (GP9)
Safety, especially for elderly patients	*“It’s unsafe to allow elderly patients to do these exercises alone at home. They may hurt themselves and make the conditions worse”* (GP12)

Almost all GPs would prefer an e-health app that was independent from the pharmaceutical industry. Some could not see themselves *“offering patients something done by a lab”* (GP16) since it was unlikely the labs were *“doing this for philanthropic reasons”* (GP12). Furthermore, some GPs felt uncomfortable about lumbar support belts being promoted in “Mon Coach Dos”. Other GPs were concerned about the fact that “*we don’t really know where the personal data is going”* (GP6) or *“if there are studies comparing simple advice with digital coaching”* (GP6). However, some GPs felt they could offer these apps to some patients *“who have good judgement”* (GP16): *“The idea is good, and I may keep it in mind, but I would spontaneously go for the health insurance application. I find it hard to see myself as a doctor offering them something made by a lab. But I think that I could tell some people. Those who will be able to discriminate well, I think I could tell them that there is another one but that it is private”* (GP16).

## Discussion

This is one of the first studies to assess the content of PA advice GPs provide in the context of low back pain. It revealed that GPs use a patient-centred approach to PA advice for low back pain with the main goal being to enable patients to participate in their care. Advice was almost always general with little information about duration and frequency but emphasising the importance of regularly performing patient-appropriate and easily achievable activities such as walking. The main barrier to providing PA advice was that GPs did not feel sufficiently trained and relied on their personal knowledge and experience without any specific support material [21]. GPs felt physiotherapists were more capable and better trained to provide PA advice.

Participants knew little about mobile e-health apps but were in favour of using this tool with younger patients. The GP perceptions of barriers to using e-health apps that patients may face were varied and included patients being unable to use technologies, poor internet connection in some regions and the lack of supervision meaning patients could injure themselves.

As has been found in the literature, our study shows that GPs’ advice about PA for low back pain was based particular on walking [17, 22]. Walking is indeed an important focus of current initiatives to encourage PA to improve health. For example, the WHO launched large scale campaigns such as “Walk the Talk” in Switzerland which had 4000 participants in 2018. The “Let’s be active: Everyone, Everywhere, Everyday” campaign was then launched in Portugal to encourage governments and authorities to make it easier for people to exercise and stay healthy [23, 24]. In France, the development of PA is supported in particular by the health sports centres (Maisons Sport Santé) [25].

Furthermore, literature agrees that GP personal experience influences the advice they provide [26, 27]. The barriers found in this study echo those found in the literature. Patient motivation is a commonly reported barrier [17, 22] and improving motivation seems essential to get patients exercising regularly. Providing patient-centred advice therefore appears an important factor to achieve this objective. Furthermore, motivational interviewing could be beneficial and has been shown to significantly improve PA outcomes in type II diabetes [28]. GP-related barriers to providing PA advice such as lack of training and lack of time, are well-documented and consistent with our findings [17, 22, 26, 29]. This results in GPs quickly referring patients to other professionals rather than giving advice themselves. Referral is most often to a physiotherapist [20, 30, 31] which is consistent with findings in this study. This follows the 2019 HAS recommendations, which are based on international recommendations [4]. Physiotherapist referral could support PA uptake because physiotherapists have an essential role in educating patients and teaching the exercises which must be continued at home. However, recommendations in Belgium, the United Kingdom and the United States mention performing exercises supervised by healthcare professionals or within a group but do not specify physiotherapists [5, 32, 33]. Furthermore, there is little literature concerning the role of physiotherapists in PA advice. Some articles state that physiotherapists would be a more legitimate healthcare professional than GPs to provide PA advice and could devote more time to it [20, 30, 31].

We found that GPs knew very little about mobile e-health apps but they felt that they could be a useful additional support and a potential interim solution whilst waiting for a physiotherapy appointment. Although digital technologies are an integral part of the WHO global action plan [24], participating GPs felt they are only appropriate for younger ‘connected’ patients. Digital tools have been launched to promote PA in the general population such as the “Jooay” app in Canada [34] but, there is little research into these apps, particularly with low back pain. However, a recent meta-analysis revealed that smartphone apps can increase PA, particularly in the short term [35]. There is also a paucity of research into apps specifically for low back pain self-management but the current evidence for using apps without supported care is inconclusive [36]. Digital tools for therapeutic patient education are available for other conditions such as diabetes and enable patients to easily view glucose measurements, meals and medications while receiving feedback on their progress. For example, the Glooko app in the USA was shown to successfully improve blood sugar levels [37]. However, despite the emergence of apps, literature reveals that physicians rarely use or recommended them [38–40]. Physicians are more likely to recommend apps after testing them and receiving positive feedback from patients, but this is still a difficult step to make. Furthermore, participating GPs would prefer an app independent from the pharmaceutical industry partners.

Further research is required into the use of New Technologies for Information and Communication (NTIC) in general medicine, such as apps to promote PA in low back pain. A clinical study evaluating the practical use of these apps could improve understanding about barriers and facilitators thus improving their use.

### Strengths and weaknesses of the study

The study strengths include the fact that purposive sampling provided the desired participant diversity. Two researchers conducted the interviews meaning more interviews could be performed thus increasing the quality of the study. Investigator subjectivity was reduced through the double reading and double coding. For the convenience of the participants, interviews were conducted at their practice. This undoubtedly increased the number of participants but was also a source of interruption during the interviews due to telephone calls and receptionists asking questions. This may have affected the content and completeness of their answers. However, the sample size remains small and therefore has an impact on the generalisability of the results.

Double coding and the use of a third researcher reduced interpretation and confirmation bias.

The COREQ criteria were followed throughout the study.

## Conclusion

GPs tend to provide general patient-centred PA advice to encourage patients with low back pain to exercise and participate in their care. Referral to physiotherapists was used to reinforce regular physical activity, maintain motivation, and improve patient adherence through supervision and follow-up. Participants admitted knowing little about e-health apps but could envisage using them with younger patients. The factors favouring and limiting both the promotion and implementation of e-health apps by GPs and use by patients need to be identified. It will then be possible evaluate apps using a comparative testing.

### Electronic supplementary material

Below is the link to the electronic supplementary material.

Supplementary Material 1

Supplementary Material 2

## Data Availability

The datasets analysed during the current study are not publicly available due to the large volume of interviews and dates on NVivo© but are available from the corresponding author on reasonable request.

## Abbreviations

GP: General Practitioner
HAS: National Health Authority
PA: Physical Activity
