# Supplementary material for: Physical activity advice given by French general practitioners for low back pain and the role of digital e-health applications: a qualitative study
Source: BMC Prim Care. 2024 Jan 29;25:44. doi: 10.1186/s12875-024-02284-w (PMC10823670; doi:10.1186/s12875-024-02284-w)
Supplement: Supplementary file 1 — Supplementary Material 1 [file 12875_2024_2284_MOESM1_ESM.docx]

Information sheet about the applications (apps) used for low back pain intended for general practitioners

| **Application** | **ACTIV’DOS** | **MON COACH DOS** |
| --- | --- | --- |
| **Description** | Coaching via a programme of exercises and advice to improve lumbar strength and health. Monitors changes in low back pain. | Videos of physical exercises, instructions about back health, explanations about how to use a lumbar support belt.  Paid version available but free access to advice and exercises. |
| **Creator/ funding** | National Health Insurance Fund. | Thuasne (creator of orthopaedic equipment) developed in partnership with Clermont Ferrand University (with contribution from doctors specialising in physical medicine and rehabilitation, physiotherapists, occupational therapists and adapted physical activity (APA) trainers). |
| **Use of data** | Data can be deleted at any point. | Data anonymised. Grita Claranet stores the health data for 2 years but the user can erase it at any time. Thuasne may also process other non-health data for the purpose of sending institutional or promotional information after the user's consent. This data, containing no health information, is stored by IBM France and kept as long as the account exists. It can be deleted at any time. |
