# Supplementary material for: Physical activity advice given by French general practitioners for low back pain and the role of digital e-health applications: a qualitative study
Source: BMC Prim Care. 2024 Jan 29;25:44. doi: 10.1186/s12875-024-02284-w (PMC10823670; doi:10.1186/s12875-024-02284-w)
Supplement: Supplementary file 2 — Supplementary Material 2 [file 12875_2024_2284_MOESM2_ESM.docx]

Date:                        Duration:

No.:     Researcher:

**Interview Guide**

Thank you for agreeing to this interview.

In this study, we want to assess physical activity in patients with low back pain regardless of duration.

We firstly need to know your age, how long you have been working at your current practice, whether your practice is rural, semi-rural or urban and whether you are a University Internship Tutor.

The interview is recorded and then transcribed but remains completely anonymous. You are free to express yourself as you wish. The interview can be interrupted at any time at your request.

We will send you the results of this work if you wish.

**Questions**

1. Tell me about the last consultation you had with a patient presenting low back pain.

- *If insufficient detail:* Do you have another situation where you were more involved in giving advice about physical activity?

1. During this consultation, how did you talk about physical activity?

- *Follow-up questions:*

What information do you provide?

What support material(s) do you use?

In which situation(s) do you rarely discuss physical activity?

In which situation(s) do you think it is important to discuss physical activity?

- *If physical activity is not discussed much:* What are the reasons?

1. Which existing digital e-health applications on the subject do you know?

- *If participant knows application(s):*

How do you use them?

How do you tell your patients about them?

- *If applications are known but not used:* For what reason(s)?

At this point in the interview, show the participant the slideshow presenting the two applications (apps) (“Mon Coach dos” and “Activ’dos”).

1. What do you think of these apps?

- Why and how could you use them?
- *If no intended use:*

For what reason(s)?

What are the perceived barriers?

- Could these apps allow you to further address the issue of physical activity?

1. Do you have anything to add about physical activity in low back pain or the apps?
